# 2525 Development of human cell-based screening assays to detect subject-specific drug-response variability

**DOI:** 10.1017/cts.2018.64

**Published:** 2018-11-21

**Authors:** Francesca Stillitano, Joshua Mayourian, Jaydev Dave, Jean-Sébastien Hulot, Roger J. Hajjar

**Affiliations:** Icahn School of Medicine at Mount Sinai

## Abstract

OBJECTIVES/SPECIFIC AIMS: The goals of this study are to develop a human-based screening assay for testing individual drug reactions and investigate the mechanism underlying susceptibility to develop diLQT. METHODS/STUDY POPULATION: We derived iPSC-CMs from 10 subjects with a high sensitivity to Sotalol (high-S group) and 10 subjects with no changes in QT interval after administration of the same drug (low-S group). Multielectrode array (MEA) was used to measure field potential duration, a surrogate to the QT interval in the electrocardiogram, in iPSC-CMs under basal conditions and in response to increasing concentrations of Sotalol. Transcriptomic profiling of iPSC-CMs from high-S Versus low-S groups was performed using RNA-sequencing. A parameter sensitivity analysis was performed on the Paci *et al*. iPSC-CM mathematical model to further support the lead hits identified via RNA-sequencing. RESULTS/ANTICIPATED RESULTS: Cardiac differentiation resulted in the generation of iPSC-CMs with appropriate cardiac channel expression and response to a hERG blocker E4031. MEA recordings showed a significantly higher response to Sotalol in iPSC-CMs from high-S compared with low-S subjects. Transcriptomic profiling identified upregulation or downregulation of genes (DLG2, KCNE4, PTRF, HTR2C, CAMKV) involved in downstream regulation of cardiac repolarization and calcium handling machinery as underlying high sensitivity to Sotalol. In silico parameter sensitivity analysis corroborated transcriptomic profiling of select genes; upregulated KCNE4 and downregulated CAMKV were predicted to positively and negatively correlate with iPSC-CM action potential duration when exposed to Sotalol, respectively. DISCUSSION/SIGNIFICANCE OF IMPACT: Our findings suggest subject-specific iPSCs can be used to model functional abnormalities observed in diLQTS and offer novel insights into iPSC-based screening assays for toxic drug reactions. Success of this study may help identify key components underlying diLQT susceptibility to ultimately develop novel therapeutic agents.

